# Blood Plasma-Derived Anti-Glycan Antibodies to Sialylated and Sulfated Glycans Identify Ovarian Cancer Patients

**DOI:** 10.1371/journal.pone.0164230

**Published:** 2016-10-20

**Authors:** Tatiana Pochechueva, Alexander Chinarev, Andreas Schoetzau, André Fedier, Nicolai V. Bovin, Neville F. Hacker, Francis Jacob, Viola Heinzelmann-Schwarz

**Affiliations:** 1 Ovarian Cancer Research, Department of Biomedicine, University Hospital Basel and University of Basel, Hebelstrasse 20, 4031, Basel, Switzerland; 2 Shemyakin-Ovchinnikov Institute of Bioorganic Chemistry, Russian Academy of Sciences, ul. MIklukho-Maklaya, 16/10, 117997, Moscow, Russian Federation; 3 Royal Hospital for Women, Gynecological Cancer Centre, School of Women’s and Children’s Health, University of New South Wales, NSW 2031, Sydney, Australia; 4 Glyco-Oncology, Ovarian Cancer Research, Department of Biomedicine, University Hospital Basel and University of Basel, Hebelstrasse 20, 4031, Basel, Switzerland; 5 Hospital for Women, Department of Gynecology and Gynecological Oncology, University Hospital Basel and University of Basel, Spitalstrasse 21, 4021, Basel, Switzerland; Gustave Roussy, FRANCE

## Abstract

Altered levels of naturally occurring anti-glycan antibodies (AGA) circulating in human blood plasma are found in different pathologies including cancer. Here the levels of AGA directed against 22 negatively charged (sialylated and sulfated) glycans were assessed in high-grade serous ovarian cancer (HGSOC, n = 22) patients and benign controls (n = 31) using our previously developed suspension glycan array (SGA). Specifically, the ability of AGA to differentiate between controls and HGSOC, the most common and aggressive type of ovarian cancer with a poor outcome was determined. Results were compared to CA125, the commonly used ovarian cancer biomarker. AGA to seven glycans that significantly (*P*<0.05) differentiated between HGSOC and control were identified: AGA to top candidates SiaT_n_ and 6-OSulfo-TF (both IgM) differentiated comparably to CA125. The area under the curve (AUC) of a panel of AGA to 5 glycans (SiaT_n_, 6-OSulfo-TF, 6-OSulfo-LN, SiaLe^a^, and GM_2_) (0.878) was comparable to CA125 (0.864), but it markedly increased (0.985) when combined with CA125. AGA to SiaT_n_ and 6-OSulfo-TF were also valuable predictors for HGSOC when CA125 values appeared inconclusive, i.e. were below a certain threshold. AGA-glycan binding was in some cases isotype-dependent and sensitive to glycosidic linkage switch (α2–6 *vs*. α2–3), to sialylation, and to sulfation of the glycans. In conclusion, plasma-derived AGA to sialylated and sulfated glycans including SiaT_n_ and 6-OSulfo-TF detected by SGA present a valuable alternative to CA125 for differentiating controls from HGSOC patients and for predicting the likelihood of HGSOC, and may be potential HGSOC tumor markers.

## Introduction

Ovarian cancer and in particular high-grade serous ovarian cancer (HGSOC) is the most deadly gynecologic cancer with an overall survival rate of less than 20% [1]. The poor outcome of this malignancy results from the lack of early disease-specific symptoms and reliable tools (*e*.*g*. tumor markers) for early diagnosis and from ineffective therapy for advanced disease and limited understanding of the initiating events and early stages of cancer progression. Though the commonly employed ovarian cancer biomarker CA125 is useful for monitoring response to chemotherapy and detecting disease recurrence, it lacks sufficient sensitivity and specificity, especially for detecting early FIGO stages of the disease [2–4]. Since also the era of genomics and transcriptomics did not produced clinically used novel biomarkers that overcome the limitations of CA125 [5], the identification of new and reliable biomarkers for early diagnosis of ovarian cancer is urgently needed.

A number of studies have proposed naturally occurring antibodies to tumor- associated carbohydrate (glycan) antigens (TACA) [6–8] or to glycopeptides [6, 9] as promising biomarkers for early detection and diagnosis of various malignancies. TACA are cancer-related glycan structures attached to proteins and lipids mostly located on the cancer cell surface. The classically known TACA are sialyl-Lewis^a^ (sLe^a^), T (or TF, Thomsen-Friedenreich) antigen, and Thomsen-nouvelle antigen (T_n_). TACA are considered potential diagnostic markers since these glycan structures resemble molecular-level glycomic ‘fingerprints’ which facilitate the discrimination between healthy and diseased states or reflect tumor micro-heterogeneity: not surprising that most clinically used tumor markers are glycoproteins [10, 11].

TACA can be recognized by anti-glycan antibodies (AGA) that, as part of innate immunity system, are present in normal sera without deliberate immunization and serve for pathogen and apoptotic cell clearance [12–14]. AGA predominantly are of IgM isotype and are crucial to immune surveillance and anti-cancer defense [15, 16] and specifically bind to glycan epitopes such as blood group A/B antigens, xenoantigens, Forssman antigen, and glycolyl-neuraminic acid containing antigens [17–21]. These characteristics of AGA make them an optimal tool to investigate aberrant glycosylation, a hallmark of carcinogenesis [22–25], and to search for biomarkers.

Considerable effort has been applied to the development of multiplexed biomarker-based test platforms [26, 27]. This includes our previously developed and optimized multiplex suspension glycan array (SGA) specifically designed for high-throughput, low material and little time consuming detection of AGA from human blood plasma or other bodily fluids [28, 29]. Our array elements consist of fluorescent microspheres, regularly coated with chemically synthesized glycans of highest purity, which makes SGA a sensitive, flexible, and robust tool suitable for biomarker research [30, 31]. Using this SGA, we focused in the present study on the multiplex detection of naturally occurring AGA to sialylated and sulfated (negatively charged) glycans in the plasma of HGSOC patients and benign donors. With few exceptions (*e*.*g*. antibodies to glycolyl-neuraminic acid and to glycans bearing it as well as antibodies to SiaLe^c^ and to 4’-O-su-LacNAc [32–35]), naturally occurring human AGA to sialo- and particularly sulfo-glycans have not yet been identified nor have they been considered for detection of ovarian cancer.

The sialic acids, *N*-acetylneuraminic acid (Neu5Ac) and *N*-glycolylneuraminic acid (Neu5Gc), are the most common terminations of glycoconjugates expressed on mammalian cells or attached to secreted molecules [36]. Increased sialylation is one of the major events in cancer and leads to overexpression of sialylated TACA [37], including Lewis blood group antigens (SiaLe^x^/SiaLe^a^), sialyl Thomsen-nouvelle antigen (SiaT_n_), and ganglioside epitopes GM_2_, GD_2_, and GD_3_. Interestingly, SiaLe^a^, SiaLe^x^, SiaT_n_, and GD_3_ were found overexpressed in ovarian cancer [25, 38]. Sulfated glycans are less abundant and no data are available (apart from glycosaminoglycans) regarding ovarian cancer. Sulfation of SiaLe^x^/SiaLe^a^ is involved in cancer cell extravasation and metastasis [39].

In the present study we investigated (i) whether AGA to an extended selection of sialylated and sulfated analogues of known TACA discriminate controls from HGSOC in the SGA, (ii) whether these AGA predict HGSOC, and how the results compare to CA125.

## Materials and Methods

### Human subjects

Two groups of female donors were involved in the current study (**Table 1**). The high-grade serous ovarian cancer (HGSOC) group consisted of patients with FIGO stages I-IV, whereof two thirds (63.7%) were stage III (n = 22). The control group (n = 31) consisted of samples from patients with benign diseases (leiomyoma, cystadenoma or fibroadenoma) or women undergoing medical control due to previous history of breast cancer or harboring *BRCA1/2* mutation. Samples were collected either from patients admitted with an adnexal mass to the Gynaecological Cancer Centre of the Royal Hospital for Women, Randwick, Australia or were seen as outpatients at the Hereditary Cancer Centre of The Prince of Wales Hospital, Randwick, Australia. All patients were over the age of 18 years and were prospectively included after giving written informed consent to this study. The responsible ethical committee (Hunter Area Research Ethics 04/04/07/3.04; South Eastern Sydney Illawarra Human Research Ethics Committee (HREC)/ Australian Research Ethics Database (AURED) Ref:08/09/17/3.02) specifically approved this study. The processing of blood plasma samples was performed consistently on ice within three hours after collection as previously described [40, 41]. Aliquoted blood plasma samples were stored at -80°C until further use.

**Table 1:** Description of study cohort (age, grade, and FIGO stage). The age is represented as mean and standard deviation for both groups.

**High-grade serous ovarian cancer** (n = 22)	**Age**	60.3±10 years		
			**n**	**%**
	**Grade**	2	6	27.3
		3	16	72.7
	**Stage**	I/II	4	18.1
		III/IV	18	81.9
**Control group** (n = 31)	**Age**	55.2±9.3 years		
	Benign		18	58.1
	Control high-risk		8	25.8
	Control previous breast cancer		5	16.1

### Production of glycopolymers with end-biotin group

The suspension glycan array (SGA) is a multiplex analysis system permitting the simultaneous analysis of multiple different biomolecules and includes (i) the production of glycopolymers composed of a polyacrylamide carrier provided with end biotin groups and side-pendant glycan residues chemically synthesized or purchased, (ii) the coupling of these glycopolymers to fluorescent microbeads, (iii) the incubation of these beads with serum samples, and (iv) the measurement of the AGA hybridized to the glycopolymers (“binding”) using fluorescently labelled reporter molecules (secondary antibodies) specific for each AGA with subsequent data analysis.

To produce these glycopolymers, sialylated and sulfated glycans for the SGA were chemically synthesized (Lectinity Holdings Inc., Moscow, Russia). The glycopolymers, used for coupling to fluorescent beads, were produced as previously described [42]. The glycopolymers are composed of a polyacrylamide (PAA) carrier (number of the average polymerisation degree, n = 220) provided with end biotin groups and side-pendant glycan residues, that are statistically distributed along the polymer backbone. The content of monomer units with glycan substitution is 20 mol%. Non-glycosylated monomer units are substituted with ethanolamine residues. Aminopropyl-GM_1a_ GM_2_ and GD_3_ glycans were purchased from Elicityl (Crolles, France) and then used for synthesis of end-biotinylated glycopolymers as described above. Hetero-bifunctional polyethylene glycol (biot–PEG_23_–NH_2_, individual compound, MW = 1300) was purchased (Iris Biotech GmbH, Marktredwitz, Germany).

### Coupling of glycopolymers to modified fluorescent microbeads

Biotinylated glycopolymers were coupled to fluorescent Bio-Plex Pro™ Magnetic COOH beads of 6.5 μm diameter with distinct spectral “addresses” (Bio-Rad Laboratories Inc., Cressier, Switzerland). Each bead’s region was embedded with a precise ratio of red and infrared fluorescent dyes allowing its identification using Bio-Plex 200 Suspension Array System (Bio-Rad Laboratories Inc.). Coupling of biotinylated glycopolymers to microbeads incorporating biot–PEG_23_–NH_2_ microbead modification was accomplished as described previously [29]. Briefly, the stock vial of microspheres (1.25×10^7^ microspheres/mL) was vigorously vortexed for 30 sec and sonicated for 15 sec in a water bath prior to its use. The tube with bead suspension (1 scale reaction: 100 μl; 1.25×10^6^ microspheres) was placed into a magnetic separator (DynaMag™-2, Life Technologies, Zug, Switzerland) for 30–60 sec and the supernatant carefully removed. The pellet was resuspended in bead wash buffer (100 μl; Bio-Plex amine coupling kit, Bio-Rad Laboratories Inc.) by vortexing and sonication, and applied for magnetic separation as described above. After gentle removal of supernatant, the pellet was resuspended in 80 μl of bead activation buffer (Bio-Plex amine coupling kit), vortexed and sonicated. Sulfo-*N*-hydroxysuccinimide sodium salt (S-NHS) and 1-ethyl-3-3,3-dimethylaminopropyl carbodiimide hydrochloride (EDC) (Thermo Fisher Scientific, Reinach, Switzerland, both 50 mg/mL in activation buffer), were prepared immediately prior to use, and 10 μl of each solution was added to the bead suspension, followed by vortexing for 30 sec. Beads were agitated in dark on rotator at room temperature for 20 min. The activated beads were applied for magnetic separation and supernatant removed. Bead pellets were resuspended in 150 μl of biot–PEG_m_–NH_2_ solution (10 mg/ml, 0.1 M NaHCO_3_, pH 8.3), agitated in dark on rotator at room temperature for 2 h. Obtained biotinylated and PEGylated beads were pelleted by magnetic separation and resuspended in 150 μl of 50 mM ethanolamine (Sigma-Aldrich Chemie GmbH, Buchs, Switzerland) in 0.1 M NaHCO_3_, pH 9.0 to quench unbound activated groups. Beads were agitated in dark on rotator at room temperature for 30 min. After magnetic separation the pellet was washed twice with 500 μl PBS, pH 7.4 and resuspended in streptavidin-solution (400 pmol streptavidin in 150 μl PBS; Bio-Rad Laboratories Inc.). Suspended beads were vortexed and agitated in dark on rotator at room temperature for 2 h. Beads were washed twice with 500 μl PBS using magnetic separator. Glycan-PAA-biot_1_ solutions (20 pmol per 1 scale coupling reaction in 150 μl PBS) were added to the reaction tubes with streptavidin-coated beads. The mixture, protected from light, was agitated on rotator at room temperature for 6 h or overnight at 4°C. Modified microspheres were applied to magnetic separator, supernatant removed and beads washed twice with 500 μl of bead storage buffer (Bio-Plex amine coupling kit). Beads were resuspended in 100 μl of bead storage buffer and concentration determined using a hemocytometer (Roth AG, Karlsruhe, Germany) before storing at 4°C, protected from light until use.

### Suspension glycan array

The Bio-Plex 200 Suspension Array System is a multiplex analysis system that permits the simultaneous analysis of up to 200 different biomolecules in a single microwell plate. The constituents of each well are drawn up into the flow-based Bio-Plex array reader, which quantifies each specific reaction based on its bead color using fluorescently labelled reporter molecules specific for each target protein followed by Bio-Plax Manager software data analysis.

Incubation buffer (PBS, pH 7.2, 1% BSA (w/v), Sigma-Aldrich Chemise GmbH) incorporating 2500 beads of each region per well (50 μl/well) was added to a Bio-Plex Pro 96-well flat bottom microplate (Bio-Rad Laboratories Inc.). The plate was washed twice with washing buffer (PBS-0.02% (v/v) Tween-20, pH 7.2) using a Bio-Plex Pro II Wash Station (Bio-Rad Laboratories Inc.). Human plasma samples, diluted 1/40, or incubation buffer alone as a control were added to wells (50 μl/well) and vigorously agitated for 30 sec on a microplate shaker before incubation on a shaker with medium speed for 1 h at room temperature in the dark. After incubation, the plate was washed thrice with washing buffer using the Bio-Plex Wash Station. Secondary antibodies (goat anti-human IgM and IgG antibodies conjugated to R-phycoerythrin (R-PE) (BioConcept, Allschwil, Switzerland), 25 ng/well in incubation buffer, 50 μl/well or incubation buffer alone as a control were added and incubated for 30 min on the plate shaker in the dark. The plate was washed thrice with washing buffer; beads were resuspended and shaken for 30 sec vigorously in 125 μl of washing buffer before being analyzed on the Bio-Plex array reader. Data were acquired in real time, analyzing 100 beads by their MFI (median fluorescence intensity) using computer software package (Bio-Plex Manager 5.1).

To obtain reliable data following experimental setups and controls were incorporated. SGA experiments were performed in duplicates three times in an independent manner. The same four control plasma samples were used in every run. The opened reduced form of D-glucose (aminoglucitol), which is not present as a structural component of regular glycosylation [30] was used to determine the background binding. In concordance with our previous data antibody binding to aminoglucitol was uniformly very low with a median of 52 MFI. This value plus two standard deviations (100 MFI) was selected as an internal experimental cut-off defining lower values as background (negative AGA reactivity). Considering recommendations from the literature for immunoassays antibody binding levels above 1000 MFI (>10 cut offs) were defined as high positive, from 500 to 1000 MFI (5 to 10 cut offs) as intermediate positive, from 100 to 500 MFI (1 to 5 cut-offs) as low positive [43, 44].

### Multiplexing of SGA

The multiplexing of SGA was performed by mixing several types of glycan-coupled microbeads in one reaction sample. For multiplex assays glycans with different structural motifs were combined in three sets (**S1 Table**) based on the absence of cross-reactivity among correspondent human plasma AGAs tested in preliminary experiments. Cross-reactivity was evaluated by comparing data obtained in singleplex (each single type of glycan-coupled microbeads in separate reaction sample) and multiplex settings using eight randomly chosen human plasma samples. The mean % coefficient of variation (CV) obtained for the same glycan in singleplex and multiplex settings did not exceed SGA inter-assay variation, described previously [28] and were smaller than 12%, thus these three sets were used for further multiplex SGA experiments.

### CA125 ELISA

The quantitative measurement of CA125 (units/ml) in blood plasma samples was performed by Abcam’s CA125 *in vitro* SimpleStep ELISA^TM^ Kit (ab195213, Abcam plc, Cambridge, UK) according to the provided protocol. Initial dilutions of plasma samples were 1/5 for control and 1/10 for HGSOC. The samples generating values out of the range for reliable determination of CA125 concentration were rescreened with adjusted dilutions.

### Statistical Methods

In a first step, descriptive statistics including median, interquartile range (IQR) and *P*-values based on Mann-Whitney U-Test are presented across study groups (univariate) and were graphically displayed. *P*-values < 0.05 were considered statistically significant. Prior to further evaluation, raw data (obtained as median fluorescence intensities, MFI) were log10-transformed to achieve approximate normal distribution.

In a second step, discriminating variables between study groups (here: anti-glycan antibodies, AGA) were assessed. L1 penalized logistic regression was performed using the package “glmnet” within the package "caret" implemented in the statistical software R [45, 46]. In our situation the "elastic-net" variant penalization of logistic regression was performed. This penalization depends on the two parameters alpha and lambda, where alpha was fixed at 0.975 and lambda was chosen using 5-fold cross-validation (with 100 repeats) to prevent from overfitting [45, 47]. The parameter lambda was additionally tuned to optimize the area under the curve (AUC), which also means that the discrimination was optimized. Details are described in https://web.stanford.edu/~hastie/Papers/Glmnet_Vignette.pdf and especially for the choice of the fixed parameter alpha. The advantage of the "elastic-net" is to perform feature selection and discrimination in one step. Additionally a “Random forest” (packages: “ROCR” and “randomForest”, [48, 49]) was applied to compare with the elastic-net. In order to tune "rf" and the "elastic-net" the package "caret" was used. This is a suitable tool to tune and compare several "machine learning" algorithm. Details are described in the corresponding package vignette.

Subsequent ROC curves with corresponding AUC's were presented and compared within the framework of "caret" [50]. Due to missing values at random (MAR) in several variables, data were imputed using the R package “mice” which implements multivariate imputation by chained equations; further details are described in van Buuren *et al*., 2011 [51].

As an exploratory tool, a tree-based model with the most important predictors and CA125 is performed to visualize potential interactions between variables [52]. This model was performed using the package “party” within R [53]. Correlations between antibodies were visualized using a heatmap with corresponding clustering based on non-parametric Pearson correlation. Statistical analysis was performed utilizing GraphPad Prism 6 software and open source statistical programming language R version 3.1.3 (https://cran.r-project.org).

## Results

### Human plasma contains anti-glycan antibodies directed to several sialylated and sulfated glycans

We and others have previously reported that human plasma contains a set of AGA directed against a variety glycans including known TACA [7, 13, 54–56]. We extended this panel of glycans and investigated whether human plasma contained AGA against closely related structural analogues and sialylated and sulfated glycans including some of which have not yet been associated with cancer. To this aim, we investigated the binding of plasma-derived antibodies from each individual across the entire cohort (control group = 31, HGSOC = 22; **Table 1**) to 22 sia- and sulfo-glycans with separate detection of AGA of the IgM-and IgG-isotype using our previously modified SGA [29].

**Fig 1:**
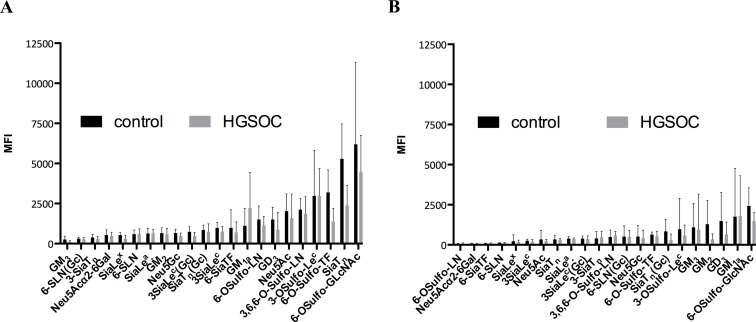
IgM AGA bind to glycans with higher reactivity than IgG AGA. Bar graph presentation of the binding (MFI; median fluorescence intensity) of AGA derived from plasma of controls (black) and HGSOC patients (grey) to each of the 22 glycans determined by SGA. Glycans are sorted by order of ascending MFI for controls. Results are separated into IgM- (**A**) and IgG- (**B**) isotypes. Error bars represent interquartile range (IQR).

The SGA results presented (**Fig 1**) showed generally higher glycan-antibody binding intensities (expressed as median fluorescence intensity, MFI) for IgM- compared to IgG isotype AGA: exceptions are anti-GM_3_, anti-6-SLN(Gc), and 3’siaT_n_. 6-O-Sulfo-GlcNAc displayed the highest AGA binding for both isotypes (MFI>1000), followed by AGA against SiaT_n_ (IgM), 6-OSulfo-TF (IgM), and 3’-O-Sulfo-Le^c^ (IgM and IgG), and by AGA against GM_3_ (IgM), 6-SLN(Gc) (IgM), Neu5Acα2-6Gal, 6-SiaTF, 6-OSulfo-LN, 6-SLN, and SiaLe^x^ (all IgG) (MFI<500). Binding intensities are usually lower in HGSOC than in control or comparable in either group for both isotypes (exception: anti-GM1_a_ IgM-isotype, which is, though not statistically significant, higher in HGSOC than in control).

### AGA to sialylated and sulfated glycans discriminate HGSOC from control with a significance comparable CA125

**Fig 2:**
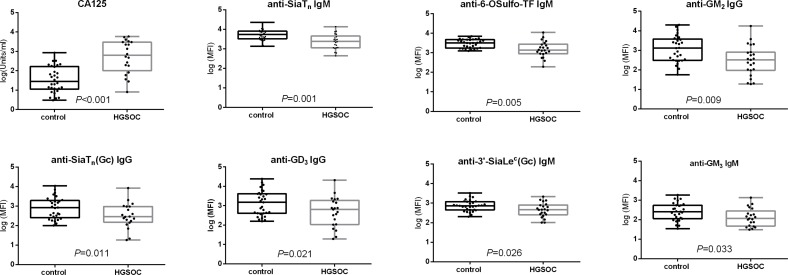
Plasma-derived AGA levels to sialylated or sulfated glycans significantly differentiate controls from HGSOC. Box-whisker plots (log transformed data) showing distribution of CA125 level and AGA binding in control and HGSOC. The significant difference between two groups is indicated by *P* values (defined by Mann-Whitney test).

We determined whether one or more of these 22 glycans have a “discriminatory value”, i.e. whether plasma-derived AGA directed against one or more of these glycans can discriminate between controls and HGSOC. The plasma level of CA125, the today most commonly used tumor marker, was included as a reference. The results are presented in **Fig 2**. As expected, CA125 discriminated controls from HGSOC with high significance (*P*<0.001). Interestingly, AGA against 7 out of 22 glycans highly significantly discriminated controls from HGSOC (*P*<0.001): anti-SiaT_n_ (IgM), anti-6-OSulfo-TF (IgM), anti-GM_2_ (IgG), anti-SiaT_n_(Gc) (IgG), anti-GD_3_ (IgG), anti-3’SiaLe^c^(Gc) (IgM), and anti-GM_3_ (IgM) (in order of descending significance based on the descriptive statistics by the magnitude of the *P*-value). Detailed listing of all glycans (grouped into mono-, di-, tri-, tetra-, and pentasaccharides) and the significance of discrimination between HGSOC and controls based on AGA binding is given in **S2 Table**.

In order to define the “most relevant” predictors (normalized to a standard deviation of 1) an L1-penalized logistic regression was calculated (feature selection = selection of predictors to discriminate between control and HGSOC). Thus, the obtained regression coefficients are directly comparable among each other. These values (**Table 2**) show that CA125 (1.27) was the “best predictor”, followed by anti-SiaT_n_ IgM (-0.75), anti-O-Sulfo-TF IgM (-0.25), anti-6-OSulfo-LN IgG (0.13), anti-SiaLe^a^ IgG (-0.03), anti-GM_2_ IgG (-0.02).

**Table 2:** Regression coefficient was obtained by L1-penalized logistic regression (details are described in Material and Methods) and sorted by descending absolute values. Elastic-net parameters alpha was set to 0.975 and lambda was estimated as 0.075. Coefficients were calculated with standardized variables (STDV = 1).

	Regression coefficient
CA125	1.27
anti-SiaT_n_ IgM	-0.75
anti-O-Sulfo-TF IgM	-0.25
anti-6-OSulfo-LN IgG	0.13
anti-SiaLe^a^ IgG	-0.03
anti-GM_2_ IgG	-0.02

Of note, not only candidates with *P*<0.05 were selected by penalized logistic regression but also two other candidates (6-OSulfo-LN (IgG) and SiaLe^a^ (IgG)). The results obtained from the penalized logistic regression were verified by “Random forest” (rf) as an independent popular algorithm for feature selection. The rf algorithm revealed CA125, anti-SiaT_n_ (IgM), anti-6-OSulfo-TF (IgM) (order of descending magnitude of significance) as the most relevant predictors, a finding that supports the stability of the selection made (S1 Appendix).

**Fig 3:**
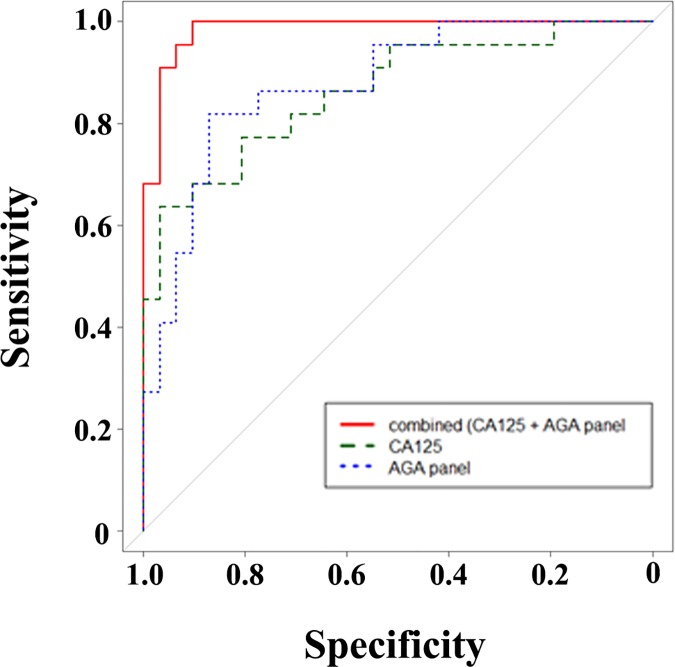
Combination of AGA and CA125 improves the diagnostic performance. ROC curves for separate and combined classifiers: CA125 alone (green), AGA panel (anti-SiaT_n_ IgM, anti-6-OSulfo-TF IgM, anti-6-OSulfo-LN IgG, anti-SiaLe^a^ IgG, and anti-GM_2_ IgG; blue), and combination of CA125 and AGA panel (red).

Finally, ROC (receiver operating characteristic) curves and corresponding AUC (area under the curve) values were calculated based on the penalized logistic regression for CA125 alone, for a panel of selected AGA based on the feature selection, and for the combination of CA125 and the panel of selected AGA to predict the diagnostic performance of this setup (**Fig 3**). The respective AUC values were 0.8644 (CA125), 0.8783 (AGA panel) and 0.9853 (combination of CA125 and AGA panel), indicating that the combination of CA125 and AGA panel (AUC of nearly 1.0) exhibited a very strong diagnostic performance for this prediction. The diagnostic performance was validated by 5-fold cross-validation revealing a significantly increasing AUC value by a combination of CA125 together with individual or both AGA (anti-SiaT_n_ (IgM), anti-6-OSulfo-TF (IgM)) (S2 Appendix).

**Fig 4:**
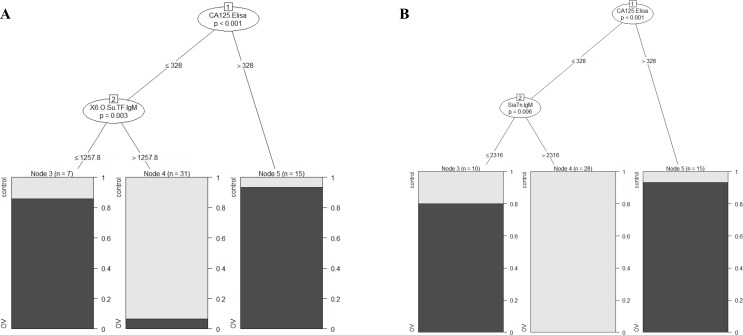
IgM to SiaT_n_ and 6-OSulfo-TF predict likelihood for HGSOC. Tree based model with CA125 and SiaT_n_ IgM (**A**) or 6-OSulfo-TF IgM (**B**). Control (light), HGSOC (dark).

A tree-based model (displays potential interactions between predictors) with CA125 and the two best predictors (anti-SiaT_n_ (IgM) or anti-6-OSulfo-TF (IgM)) was computed (**Fig 4**). This model provided an accurate likelihood to predict HGSOC in individuals with CA125 serum levels of less than a certain value. Our computation for this cohort proposes a 90% likelihood for HGSOC, if CA125 serum values are higher than 328 units/ml. For values lower than 328 units/ml, levels of anti-SiaT_n_ or anti-6-OSulfo-TF rather than CA125 values are accurate predictors for HGSOC: here anti-SiaT_n_ MFI values lover than 2316 predict an 80% likelihood for HGSOC (**Fig 4A**) and accordingly an 85% likelihood for HGSOC for anti-6-OSulfo-TF values less than 1258 (**Fig 4B**). These results demonstrate that, apart from CA125, IgM-isotype AGA against SiaT_n_ and O-Sulfo-TF are highly significant predictors for HGSOC.

### Structural and chemical modification of sialo- and sulfo-glycans can affect anti-glycan binding

We observed that binding to SiaT_n_ is different for IgM-isotype (discriminating) and for IgG-isotype (not discriminating) AGA and that IgM-isotype AGA against SiaT_n_ discriminated between control and HGSOC but its analogue 3-SiaT_n_ did not (**Fig 1**). SiaT_n_ and 3-SiaT_n_ differ in their glycosidic linkage (α2–6 *vs*. α2–3), indicating that the type of this glycosidic linkage “decides” between discriminating or non-discriminating, i.e. the discrimination is lost when SiaT_n_ (α2–6) is replaced by 3-SiaT_n_ (α2–3). These observations suggest that AGA-glycan binding is a fine-tuned matter worthwhile looking into it in more detail (**Fig 1**). The substitution of Neu5Ac in SiaT_n_ by NeuGc in SiaT_n_(Gc) decreased IgM AGA binding in both groups and increased IgG binding in the control group and abrogated the discrimination of control and HGSOC for the IgM-isotype but produced discrimination for IgG-isotype. Likewise, the substitution of Neu5Ac in 3’SiaLe^c^ by a sulfo-group in 3’-OSulfo-Le^c^ increased in immunoreactivity of both, the IgM- and the IgG-isotype AGA, without affecting the non-discrimination. Likewise, substitution of Neu5Ac in 3’SiaLe^c^ by non-human Neu5Gc slightly decreased IgM immunoreactivity and produced discrimination between control and HGSOC (*P* = 0.026) whereas it slightly increased IgG immunoreactivity without affecting non-discrimination. AGA binding to 6-OSulfo-TF was higher than to its sialylated-analogue 6-Sia-TF for both isotypes, and anti-6-OSulfo-TF IgM highly significantly discriminated between control and HGSOC (*P* = 0.005) while this discrimination was lost for 6-Sia-TF. While uncharged TF (Galβ1-3GalNAcα) did not, its sulfated analogue 6-OSulfo highly significantly (*P* = 0.002) discriminated between the two groups (**S1 Fig**), indicating that the observed discrimination of anti-6-OSulfo-TF was attributed to the sulfation of TF antigen.

A correlation matrix of all AGA for control and HGSOC was calculated. The resulting dendrogrammed heatmap computed by Pearson correlation analysis (**S2 Fig**) identified two major clusters: one comprising mostly IgM AGA and one mostly IgG AGA. While discriminating AGA against GM_2_ (IgG), SiaT_n_(Gc) (IgG), GD_3_ (IgG), 3’SiaLe^c^(Gc) (IgM), and GM_3_ (IgM) belonged to definite clusters, our two top discriminating candidates anti- SiaTn (IgM) and anti-6-OSulfo-TF (IgM) were at the margin of the IgM cluster (S3 Appendix). CA125 positively correlated with the IgM rather than the IgG cluster in HGSOC and control, indicating a given likelihood that higher CA125 values mean higher MFI values for IgM AGA binding in both groups.

## Discussion

The potential of plasma-derived AGA against an extended selection of structural and chemical analogues of known TACA to discriminate benign controls from HGSOC patients in the SGA and the ability of these AGA to predict HGSOC just from their levels in the plasma were investigated and compared to CA125.

Three major findings emerged from this investigation. Firstly, AGA to seven out of the 22 investigated glycans significantly differentiated plasma samples from control women and HGSOC patients in the SGA: the two top AGA, anti-SiaT_n_ and anti-6-OSulfo-TF, and CA125 (standard tumor marker commonly used in the clinics) distinguished with comparable power. Secondly, the predictive value (AUC) of the 5 top candidate AGA together (anti-SiaT_n_, anti-6-OSulfo-TF, anti anti-6-OSulfo-LN, anti-SiaLe^a^, and anti-GM_2_) is comparable to CA125, but was markedly higher (nearly 1.0) if combined with CA125. Thirdly, SiaT_n_ and 6-OSulfo-TF were also valuable predictors for HGSOC when CA125 values were inconclusive, i.e. below a certain threshold. Another finding was that the AGA-glycan binding in the SGA was in some cases antibody isotype-dependent or sensitive to a switch in the glycosidic linkage and even to minor chemical modification in the glycans. The following conclusion may be drawn: Plasma-derived AGA to particular glycans (*e*.*g*. SiaT_n_ and 6-OSulfo-TF) are a valuable alternative to CA125 for the discrimination of controls from HGSOC patients and for predicting the likelihood of HGSOC in a cohort, proposing these newly identified glycan-antibody interactions as potential HGSOC tumor markers in the SGA setting.

The herein identified candidate glycans may, however, at this point not replace the currently used ovarian cancer biomarker CA125 but certainly may serve as a complementary marker and predictor for HGSOC in the diagnosis of this disease. The identified candidate glycans do not belong to one specific group of glycan antigens but to gangliosides (GM_2_, GM_3_, GD_3_), Thomsen-nouvelle (SiaT_n_ and SiaT_n_(Gc)), Thomsen-Friedenreich (6-OSulfo-TF), and “Lewis” related antigen, indicating the absence of any obvious systematic or preference. Most of them and their non-modified analogues like TF and T_n_ are well-described TACA [57, 58], and some (e.g. SiaLe^a^, SiaLe^x^, SiaT_n_, GD_3_) have been shown to be overexpressed in ovarian cancer [25, 38].

We observed that even minor modifications of the glycans alter AGA binding reactivity and consequently their discriminating properties, highlighting the high specificity of (certain) AGA in the recognition of their glycan epitopes. Our finding that 6-OSulfo-TF but not its non-sulfated analogue TF, was (apart from CA125) the second-best HGSOC from control discriminator is noteworthy, because the role of glycan sulfation (in contrast to glycan sialylation) has not yet been studied in ovarian cancer. On the other hand, the discriminating property of 6-OSulfo-TF was lost with its sialylated-analogue 6-Sia-TF. The sulfation of SiaLe^x^ and SiaLe^a^ may play a role in cancer cell extravasation and metastasis, possibly by increasing their affinity to selectins [39]. In addition, the substitution of Neu5Ac by Neu5Gc in 3’SiaLe^c^ produced discrimination, whereas that of Neu5Ac in SiaT_n_ by NeuGc in SiaT_n_(Gc) abrogated discrimination between HGSOC and control groups, and the glycosidic linkage switch from α2–6 in SiaT_n_ into α2–3 in 3-SiaT_n_, respectively abrogates this discrimination. Interestingly, α2–6 (in contrast to α2–3) sialylation is a hallmark of malignant transformation [57] and a mediator of cancer progression *in vivo* [59].

The AGA-glycan binding intensities were generally lower in HGSOC than in control or comparable in either group, which is fully consistent with previous studies including ours and indicates that cancer patients commonly had lower serum AGA levels [41, 60]: the exception to this is anti-GM1_a_ IgM antibody, which is apparently elevated in HGSOC compared to controls. GM1_a_ ganglioside has been suggested to inhibit cellular proliferation and migration, and to enhance differentiation [61], but it remains unknown why the serum level AGA is elevated in HGSOC.

In regards to early detection of ovarian cancer, we have shown that AGA to sialylated and sulfated glycans compete with and improve the detection of CA125 alone. Moreover, our experimental setup allows simultaneous measurement of CA125 and selected AGA in only a minute amount of plasma samples, which would minimize the health costs. Multiplex measurement of CA125 together with other biomarkers has already been validated in a multiplexed system [27] and therefore can be measured also by SGA together with AGA in one single multiplex approach and in one sample. Despite the cross-validation applied here, additional studies on extended samples size and external validation cohorts have to be performed to confirm our findings. An improvement of the current setup might also be achieved by incorporating risk factors such as menopausal status. This has already been demonstrated to improve the diagnostic performance [62–64].

## Supporting Information

S1 Appendix Feature selection and ranking for univariate method, glmnet, and random forest. (PDF)

S2 Appendix Cross-validation of AUC values. (PDF)

S3 Appendix Spearman correlation and corresponding *p*-values of selected parameters. (PDF)

S1 Fig Box-and-whisker plot presenting distribution of IgM AGA levels directed to 6-OSulfo-TF and TF in control and HGSOC groups. Significant difference between control (black) and HGSOC (gray) is indicated (*P* values, Mann-Whitney test).
(PDF)

S2 Fig Dendrogrammed correlation matrix is shown for all AGA reactivities in color-coded representation in the control and HGSOC group separately. Heatmap is based on non-parametric Spearman correlation comparing all AGA and CA125 against each other. Red color (positive correlation), blue (negative correlation), white (no correlation). Control group (**A**), HGSOC (**B**).
(PDF)

S1 Table Sets of glycans used in multiplexed measurements in SGA. (PDF)

S2 Table Sialo-and sulfo glycans used in SGA. *P* values for comparison of HGSOC *vs*. control are defined by Mann-Whitney test on log-transformed data; FDR (False discovery Rate) adjusted *P* values less than 0.1 are shown. *P* values considered statistically significant (<0.05) are shown in red.
(PDF)

## Abbreviations

AGA: anti-glycan antibody
HGSOC: high-grade serous ovarian cancer
SGA: suspension glycan array
CA125: Cancer-Antigen 125
T_n_: Thomsen-nouvelle (antigen)
TF: Thomsen-Friedenreich (antigen)
FIGO: International Federation of Gynecology and Obstetrics
TACA: tumor-associated carbohydrate antigen
Sia: sialyc acid
Le: Lewis
Neu5Ac: acetylneuraminic acid
Neu5Gc: *N*-glycolylneuraminic acid
LN: Galβ1-4GlcNAc
SLN: Sialyl-N-acetyllactosamine
ROC: Receiver Operating Characteristic
AUC: area under the curve
PAA: polyacrylamide
PEG: polyethylene glycol
biot: biotinylated
PBS: phosphate buffered saline
R-PE: R-phycoerythrin
CV: coefficient of variation
IQR: interquartile range
