# Supplementary material for: Blood Plasma-Derived Anti-Glycan Antibodies to Sialylated and Sulfated Glycans Identify Ovarian Cancer Patients
Source: PLoS One. 2016 Oct 20;11(10):e0164230. doi: 10.1371/journal.pone.0164230 (PMC5072665; doi:10.1371/journal.pone.0164230)
Supplement: S1 Appendix — (PDF) [file pone.0164230.s001.pdf]

S1 Appendix

Feature selection and ranking for univariate method, glmnet and random forest

Univariate*	Glmnet**	Random forest***	Rank
CA125	CA125	CA125	1
anti-SiaT$_n$ IgM	anti-SiaT$_n$ IgM	anti-SiaT$_n$ IgM	2
anti-O-Sulfo-TF IgM	anti-O-Sulfo-TF IgM	anti-O-Sulfo-TF IgM	3
anti-6-OSulfo-LN IgG	anti-6-OSulfo-LN IgG	SiaLex.IgM	4
	anti-SiaLe^a IgG	SiaTn.Gc..IgG	5
	anti-GM$_2$ IgG	GM2.IgG	6
		GM1a.IgM	7

* based on significance, $p<0.05$

** based on elastic-net feature selection

*** based on random forest variable importance, only the seven best are presented.
