# Supplementary material for: Blood Plasma-Derived Anti-Glycan Antibodies to Sialylated and Sulfated Glycans Identify Ovarian Cancer Patients
Source: PLoS One. 2016 Oct 20;11(10):e0164230. doi: 10.1371/journal.pone.0164230 (PMC5072665; doi:10.1371/journal.pone.0164230)
Supplement: S2 Appendix — (PDF) [file pone.0164230.s002.pdf]

S2 Appendix

AUC values based on 5-fold cross-validations for "glmnet"

1) 5*100 cross-validations were done, a descriptive statistics of these results are presented.
2) 2-4th comparisons means given biomarkers + CA125.

AUC

	Min.	1st Qu.	Median	Mean	3rd Qu.	Max.	NA's
CA125	0.4167	0.8000	0.8964	0.8708	0.9583	1	0
SiaTn	0.6250	0.9167	0.9667	0.9518	1.0000	1	0
6.O.Su.TF	0.5833	0.8750	0.9583	0.9251	1.0000	1	0
both	0.6667	0.9405	1.0000	0.9618	1.0000	1	0

Comparisons all against all

P-value adjustment: bonferroni
Upper diagonal: estimates of the difference
Lower diagonal: *p*-value for H0: difference = 0

ROC

	CA125	SiaTn	6.O.Su.TF	both
CA125		-0.081067	-0.054326	-0.091019
SiaTn	< 2.2e-16		0.026740	-0.009952
6.O.Su.TF	< 2.2e-16	4.397e-09		-0.036693
both	< 2.2e-16	0.03235	2.138e-15	
