# Supplementary material for: Blood Plasma-Derived Anti-Glycan Antibodies to Sialylated and Sulfated Glycans Identify Ovarian Cancer Patients
Source: PLoS One. 2016 Oct 20;11(10):e0164230. doi: 10.1371/journal.pone.0164230 (PMC5072665; doi:10.1371/journal.pone.0164230)
Supplement: S3 Appendix — (PDF) [file pone.0164230.s003.pdf]

S3 Appendix

Spearman correlation with corresponding *p*-values:

Correlation:

	CA125	SiaTn.IgM	6.O.Su.TF.IgM
CA125	1.00	-0.13	-0.06
SiaTn.IgM	-0.13	1.00	0.46
6.O.Su.TF.IgM	-0.06	0.46	1.00

P-values:

	CA125	SiaTn.IgM	6.O.Su.TF.IgM
CA125		0.3510	0.6597
SiaTn.IgM	0.3510		0.0005
6.O.Su.TF.IgM	0.6597	0.0005	
