# Supplementary figures and images for: Blood Plasma-Derived Anti-Glycan Antibodies to Sialylated and Sulfated Glycans Identify Ovarian Cancer Patients
Source: PLoS One. 2016 Oct 20;11(10):e0164230. doi: 10.1371/journal.pone.0164230 (PMC5072665; doi:10.1371/journal.pone.0164230)

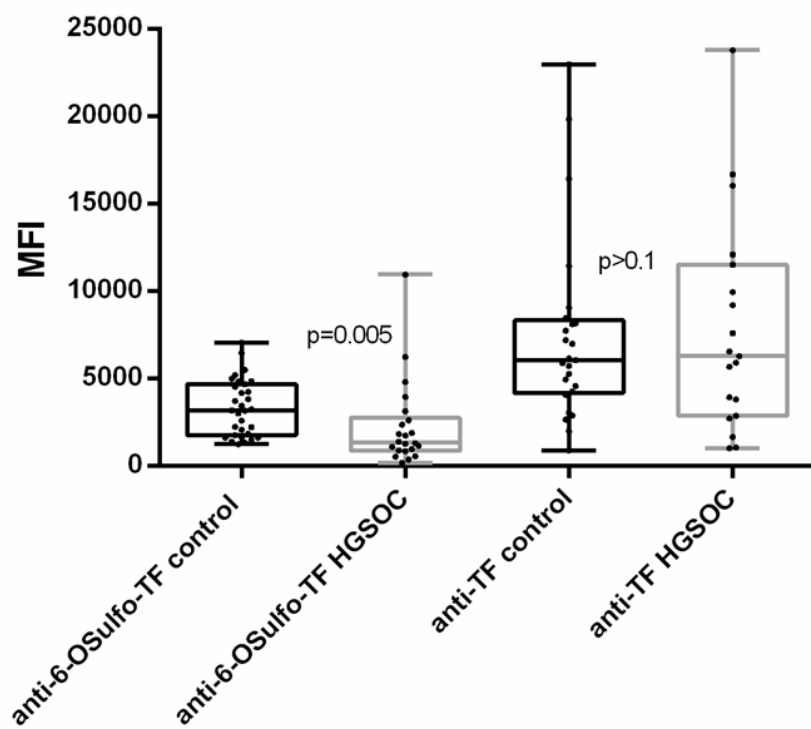

Supplement: S1 Fig — Significant difference between control (black) and HGSOC (gray) is indicated (P values, Mann-Whitney test). (PDF) [file pone.0164230.s004.pdf]

**A**

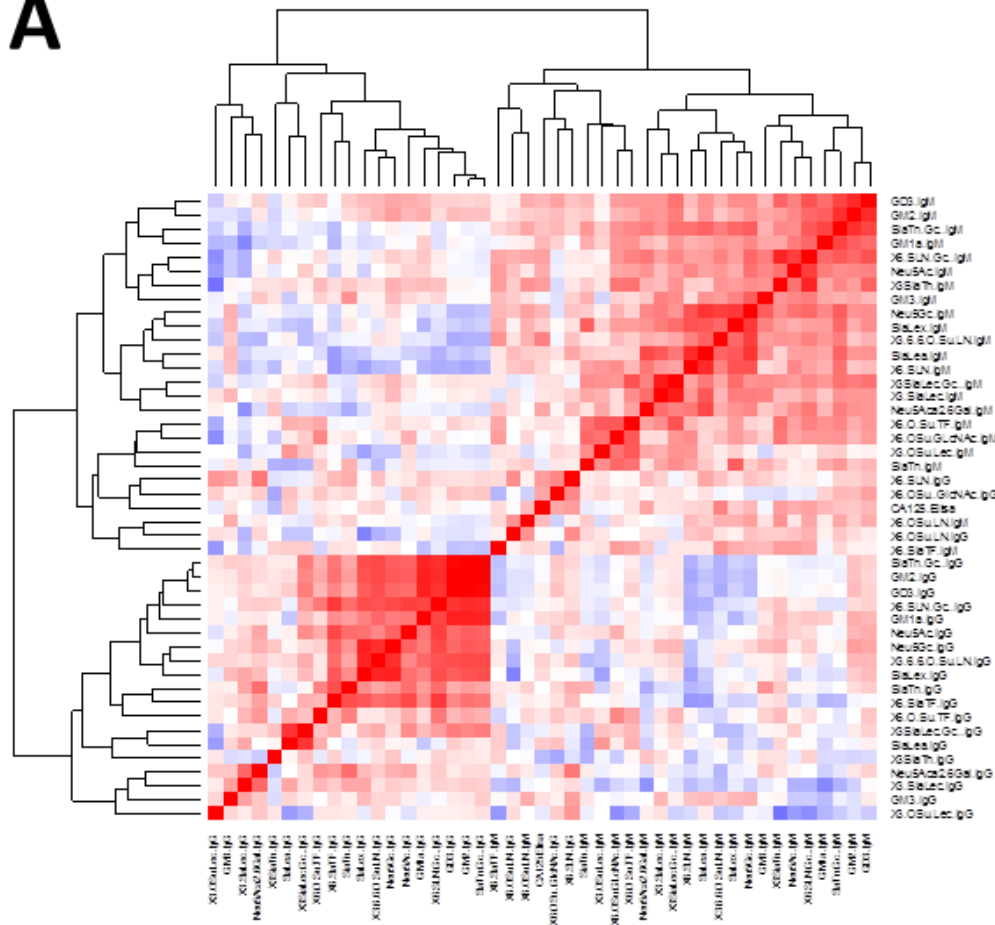

**B**

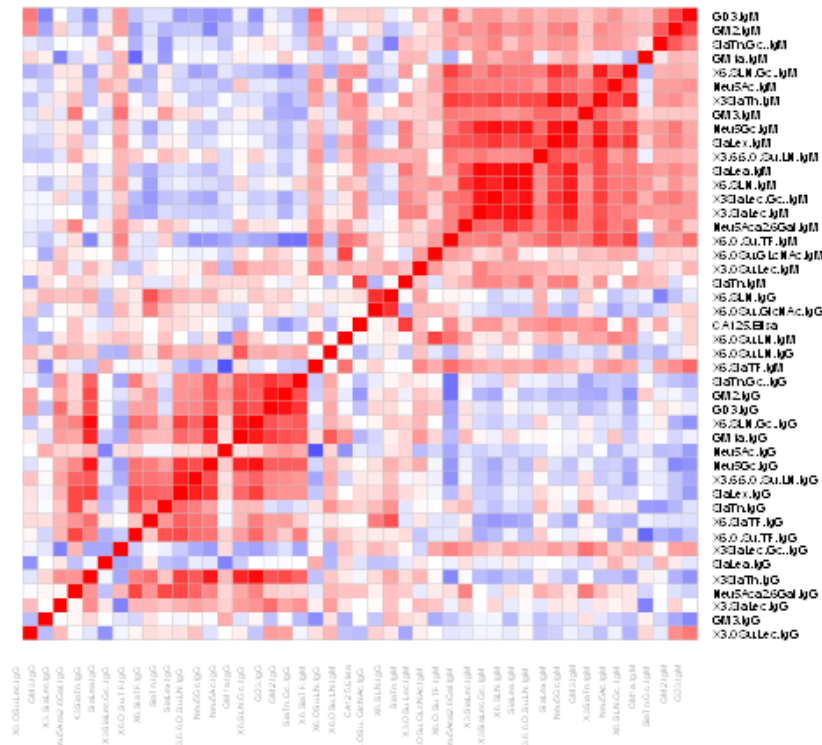

Supplement: S2 Fig — Heatmap is based on non-parametric Spearman correlation comparing all AGA and CA125 against each other. Red color (positive correlation), blue (negative correlation), white (no correlation). Control group (A), HGSOC (B). (PDF) [file pone.0164230.s005.pdf]
