# Supplementary material for: Blood Plasma-Derived Anti-Glycan Antibodies to Sialylated and Sulfated Glycans Identify Ovarian Cancer Patients
Source: PLoS One. 2016 Oct 20;11(10):e0164230. doi: 10.1371/journal.pone.0164230 (PMC5072665; doi:10.1371/journal.pone.0164230)
Supplement: S1 Table — (PDF) [file pone.0164230.s006.pdf]

**Set 1**	**Set 2**	**Set 3**
Neu5Gc	Neu5Ac	$SiaLe^x$
$3'SiaLe^c$	$SiaLe^a$	$SiaT_n$(Gc)
$SiaT_n$	Neu5Aca2-6Galβ	$3'$-OSulfo-Le^c
3'SL, GM_3	$3SiaLe^c$(Gc)	6-OSulfo-LacNAc
6-SLN(Gc)	6-SiaTF	6-O-Sulfo-TF
GD_3	6'SLN	GM_{1a}
6-OSulfo-GlcNAc	GM_2	
3,6,6'-OSulfo3-LacNAc	3-$SiaT_n$	
