# Supplementary material for: Blood Plasma-Derived Anti-Glycan Antibodies to Sialylated and Sulfated Glycans Identify Ovarian Cancer Patients
Source: PLoS One. 2016 Oct 20;11(10):e0164230. doi: 10.1371/journal.pone.0164230 (PMC5072665; doi:10.1371/journal.pone.0164230)
Supplement: S2 Table — P values for comparison of HGSOC vs. control are defined by Mann-Whitney test on log-transformed data; FDR (False discovery Rate) adjusted P values less than 0.1 are shown. P values considered statistically significant (<0.05) are shown in red. (PDF) [file pone.0164230.s007.pdf]

Glycan	Short name	Schematic structure	*P* value	
**Monosaccharides**			**IgM**	**IgG**
**Neu5Gcα-**	**Neu5Gc**		>0.1	>0.1
**Neu5Aca-**	**Neu5Ac**		>0.1	>0.1
**6-O-Su-GlcNAcβ-**	**6-OSulfo-GlcNAc**	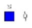	>0.1	>0.1
**Disaccharides**				
**Neu5Acα2-6GalNAcα-**	**$\mathrm{SiaT_n}$**	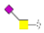	**0.001**	>0.1
**Neu5Acα2-3GalNAcα-**	**$\mathrm{3\text{-}SiaT_n}$**	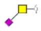	>0.1	>0.1
**Neu5Gca2-6GalNAca-**	**$\mathrm{SiaT_n(Gc)}$**	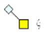	>0.1	**0.011**
**Galβ1-3(Su-6)GalNAcα-**	**6-OSulfo-TF**	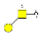	**0.005**	>0.1
**Galβ1-4(Su-6)GlcNAcβ-**	**6-OSulfo-LN**	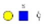	>0.1	0.064
**Su-6Galβ1-4(Su2-3,6)GlcNAcβ-**	**3,6,6'-OSulfo-LN**	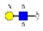	>0.1	>0.1
**Su-3Galβ1-3GlcNAcβ-**	**3'-OSulfo-$\mathrm{Le^c}$**	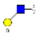	>0.1	>0.1
**Neu5Aca2-6Galβ-**		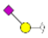	>0.1	>0.1
**Trisaccharides**				
**Neu5Aca2-6Galβ 1-4GlcNAcβ-**	**6'SLN**	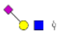	>0.1	>0.05
**Neu5Gcα2-6Galβ1-4GlcNacβ-**	**6'SLN(Gc)**	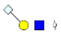	>0.1	>0.1
**Neu5Gca2-3Galβ 1-3GlcNAcβ-**	**3'$\mathrm{SiaLe^c}$(Gc)**	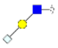	**0.026**	>0.1
**Neu5Aca2-3Galβ 1-3GlcNAcβ-**	**3'$\mathrm{SiaLe^c}$**	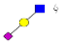	>0.1	>0.1
**Neu5Gca2-3Galβ 1-3GlcNAcβ-**	**6-SiaTF**	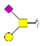	>0.1	>0.1
**Neu5Aca2-3Galβ 1-4Glcβ-**	**3'SL ($\mathrm{GM_3}$)**	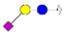	**0.033**	>0.1
**Tetrasaccharides**				
**GalNAcβ1-4(NeuAcα2-3)Galβ1-4Glcβ-**	**$\mathrm{GM_2}$**	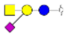	>0.1	**0.009**
**NeuAcα2-8NeuAcα2-3Galβ1-4Glcβ-**	**$\mathrm{GD_3}$**	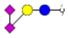	>0.1	**0.021**
**Neu5Aca2-3Galβ1-3(Fucα1-4)GlcNAcβ-**	**$\mathrm{SiaLe^a}$**	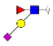	>0.1	>0.05
**Neu5Aca2-3Galβ1-4(Fucα1-3)GlcNAcβ-**	**$\mathrm{SiaLe^x}$**	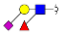	>0.05	>0.1
**Pentasaccharides**				
**Galβ1-3GalNAcβ1-4(NeuAcα2-3)Galβ1-4Glcβ-**	**$\mathrm{GM_{1a}}$**	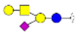	>0.1	>0.1

				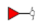		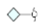
Glucose	N-acetylglucosamine	Galactose	N-acetylgalactosamine	Fucose	Acetylneuraminic acid	Glycolylneuraminic acid
